# Improved salt iodation methods for small-scale salt producers in low-resource settings in Tanzania

**DOI:** 10.1186/1471-2458-9-187

**Published:** 2009-06-17

**Authors:** Vincent D Assey, Thorkild Tylleskär, Philip B Momburi, Michael Maganga, Nicholaus V Mlingi, Marie Reilly, Ted Greiner, Stefan Peterson

**Affiliations:** 1 Food Science and Nutrition Department, Tanzania Food and Nutrition Centre (TFNC), 22 Ocean Road, PO Box 977 Dar-Es-Salaam, Tanzania; 2 Centre for International Health (CIH), University of Bergen, Årstadveien 21, N-5009 Bergen, Norway; 3 Department of Women's and Children's Health, Unit of International Maternal and Child Health (IMCH), *Akademiska Sjukhuset*, Uppsala University, SE -75185 Uppsala, Sweden; 4 Geological Survey of Tanzania, Ministry of Energy and Minerals, PO Box 903 Dodoma, Tanzania; 5 Medical Epidemiology and Biostatistics, Karolinska Institutet, Stockholm, Sweden; 6 Food and Nutrition Department, Hanyang University, 17 Haengdang-dong, Seongdong-gu Seoul 133-790, South Korea; 7 Department of Public Health Sciences, Division of International Health (IHCAR), Nobel v 9, Karolinska Institutet, S-17177, Stockholm, Sweden; 8 Makerere University School of Public Health, Kampala, Uganda

## Abstract

**Background:**

Universal salt iodation will prevent iodine deficiency disorders (IDD). Globally, salt-iodation technologies mostly target large and medium-scale salt-producers. Since most producers in low-income countries are small-scale, we examined and improved the performance of hand and knapsack-sprayers used locally in Tanzania.

**Methods:**

We studied three salt facilities on the Bagamoyo coast, investigating procedures for preparing potassium-iodate solution, salt spraying and mixing. Different concentrations of solution were prepared and tested using different iodation methods, with the aim of attaining correct and homogeneous iodine levels under real-life conditions. Levels achieved by manual mixing were compared to those achieved by machine mixing.

**Results:**

The overall median iodation level in samples of salt iodated using previously existing methods was 10.6 ppm (range 1.1 – 110.0 ppm), with much higher levels in the top than the bottom layers of the salt bags, p < 0.0001. Experimentation using knapsack-sprayers and manual mixing led to the reliable achievement of levels (60.9 ppm ± 7.4) that fell within the recommended range of 40 – 80 ppm. The improved methods yielded homogenous iodine concentrations in all layers of salt-bags (p = 0.58) with 96% of the samples (n = 45) falling within 40 – 80 ppm compared to only 9% (n = 45) before the experiment and training (p < 0.0001). For knapsack-spraying, a machine mixer improved the iodine levels and homogeneity slightly compared to manual mixing (p = 0.05).

**Conclusion:**

Supervised, standardized salt iodation procedures adapted to local circumstances can yield homogeneous iodine levels within the required range, overcoming a major obstacle to universal salt iodation.

## Background

Eliminating iodine deficiency disorders (IDD) is now considered "within grasp" [1,2]. Globally, households' use of iodated salt had increased from 10% to 70% by 2006 [3]. Accomplishing the last-mile of iodization of the remaining un-iodized salt will no doubt prove a challenge in many cases and it is the most expensive part of the business [4,5]. Nevertheless, it is worth the effort because those not covered will often prove to be those most needing it. Furthermore, even in otherwise well-nourished populations, consumption of less iodine than is required by the human bodies have serious consequences for mental and physical productivity of all populations [4]. WHO, UNICEF and the International Council for Control of Iodine Deficiency Disorders (ICCIDD) have recommended strategies for salt iodation [6-8]. Suffering from moderate to severe IDD [9,10], Tanzania adopted universal salt iodation (USI) in the 1990s as a permanent strategy for prevention and control of IDD [8,11]. Between 1992 and 1998, UNICEF and FAO procured and installed seventy-two salt iodation machines in Tanzania for large and medium scale salt producers. A 'Salt Act' was then passed by parliament prohibiting sale of non-iodated salt for human and animal consumption after January 1995. The 75 – 100 parts per million (ppm) iodation level initially recommended for salt factories was reduced to 40 – 80 ppm in 2006 [12,13]. Sensitisation and training of personnel at various levels of salt iodation focused on understanding the IDD problem and control measures, salt iodation processes and quality control at various stages during salt handling [8,11].

Spot surveys in Tanzania in 1995 revealed that iodated salt had become more available at household level and that goitre prevalence was reduced where iodated salt could be obtained [14,15]. In 1999–2000 an evaluation of the IDD control programme in 16 goitre endemic districts indicated that 83% of households were consuming iodated salt, but with highly variable iodine concentrations [12]. In 2004, Tanzania Demographic Health Surveys found that 74% of households were using iodated salt nationwide but usage in certain regions was much lower [16]. Small-scale salt producers were thought to be the likely sources of non-iodated salt, while medium/larger producers could be sources of under- and/or over- iodated salt.

In many low-income countries, small-scale salt producers operate with minimal organisation and there is little or no quality control. Small salt fields are widely scattered and do not lend themselves to government regulation [17]. In Tanzania, over 6,500 people, mainly women, are involved in this type of small-scale salt production [18], estimated to account for 20 – 40% of the total national salt production (unpublished TFNC report, 2003). These small scale salt producers usually have limited financial means and lack access to technical assistance. As a result, the salt produced is of poor quality, with impurities and exposure to high humidity that are known to cause high iodine losses while purified salt has been reported to improve stability of iodine [19].

An inventory of the salt-production sites[20] that had been supplied with salt iodation machines revealed that (1) most salt factories no longer used them owing to high running costs, (2) the capacity to repair breakdowns was minimal or non-existent and (3) cooperatives created to give many small-scale producers access to the equipment did not work. Both large- and small-scale producers were mainly using hand spray pumps and other small gadgets to spray potassium iodate solution on to the salt, producing highly variable iodine contents. In 2003, salt samples collected among the small-scale producers showed that 93% (n = 85) did not conform with the then recommended iodation levels; most were too low [20].

In order to devise improved iodation methods for small-scale production, we set out interactively to study the various ways in which the producers used hand sprayers and how iodation level and homogeneity could be improved using the technology already in use. This paper reports (1) information obtained through observations, interviews and analysis of iodine from salt samples in stock at the sites, (2) the performance of the iodate spraying methods found in use (with and without supervision), (3) tests of modified methods to improve concentration and homogeneity and (4) a comparison of manual mixing with a cement mixer.

## Methods

### Study area

The coastal Bagamoyo district, located 45 km north of Dar es Salaam city, was selected as the study area. This district has the advantage of hosting large, medium and small scale salt producers' operations, meeting the needs for the study. Three salt farms categorised as small, medium and large were studied. All use solar evaporation, drying the salt in heaps. The owners gave the study team access to their salt farms and workers to make observations of the methods/equipment used for iodation.

### Qualitative data

We observed the procedures used to make the potassium iodate (KIO_3_) solution, to mix it with salt, and to estimate the size of the salt heaps and thus the amount of iodate to be added. We conducted unstructured qualitative interviews with production site managers and salt workers about the rationale of each step in the observed iodation procedure and the practicability of possible modifications [21].

### Sample collection and analysis

We sampled 50 kg bags of recently-produced salt and then tested several procedural modifications. In each experiment, one to three typical heaps of salt found on the salt evaporation-pan were used. Typical heaps fill 20 – 40 fifty-kg bags. The final size of the heap in tonnes was estimated by counting the final number of sacks. From each heap, five sacks were randomly selected and a total of 15 salt samples were taken – one from each of the top, middle and bottom layers in each sack. A total of 225 salt samples were collected in the different experiments and were stored in 200 g capacity containers with airtight stoppers. They were initially tested by a rapid test kit (RTK) – a semi-quantitative method to ensure the presence of iodine [22], then for iodine concentration using the recommended iodometric method [7,23] at the Tanzania Food and Nutrition Centre (TFNC), the National Iodine Reference Laboratory based in Dar es Salaam. The coefficient of variation (CV) of 3.6% (n = 16, mean 64.4 ± 2.3) obtained is far below the 15% that is considered good performance [23].

### Assessment of existing iodation methods

**Figure 1:**
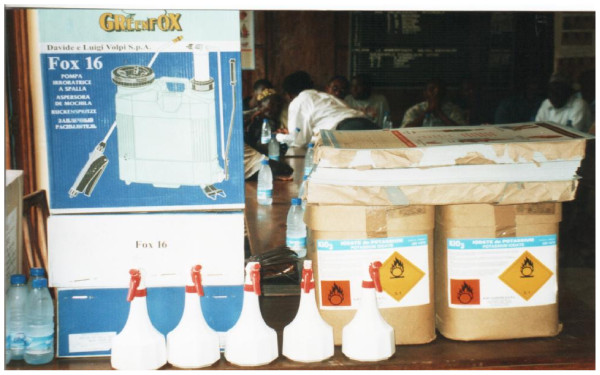
**Essential equipment: knapsack sprayer, hand sprayers (white containers with red nozzle), KIO_3 _and instructional literature for small scale salt iodation**.

Two main manual iodation methods were found in use: knapsack/backpack sprayers (20 litre capacity) and hand-bottle sprayers (one-litre capacity), Figure 1. These were assessed for uniformity in achieving the recommended salt iodation levels.

### Development of an improved iodation method

The potassium iodate in use (KIO_3_, molar weight = 214.9 g/mol, estimated cost $18/kg) originated from the INQUIM Company in Chile was purchased through UNICEF. It conformed to U.S. Food Chemical Codex specifications [7].

Different KIO_3 _concentrations were prepared on the basis of the experience gained from participant observations, interviews with salt workers and the iodine levels obtained from baseline salt sample analyses. It was determined that the correct quantity of KIO_3 _to be dissolved in 20 litres of fresh water is 204 g (conversion factor 1.685 ppm (mg/kg) KIO_3 _= 1 ppm (mg/kg) iodine). This should result in an iodine level of approximately 60.5 ppm (± 15 SD) after spraying the heaps three times. The correct quantity of solution to be sprayed on each heap was determined according to an estimate of heap size, spraying techniques and mixing procedures and was guided by repeated sampling of the iodine concentration in the salt. This procedure was repeated until an optimum uniformity of salt iodine concentration was achieved. A mark was made on the knapsack sprayer showing the volume of KIO_3 _solution used.

**Figure 2:**
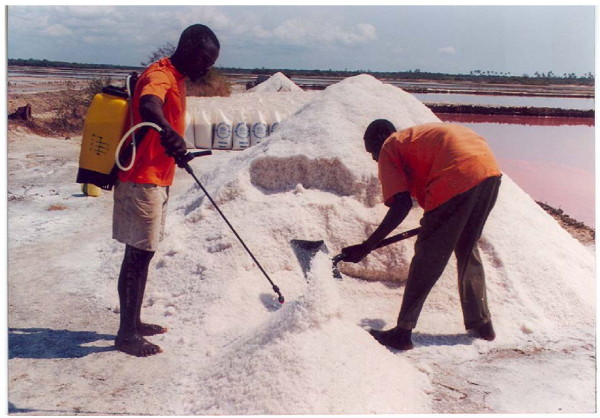
**Iodation of salt using a knapsack sprayer and manual mixing of a 2-ton salt heap**.

**Figure 3:**
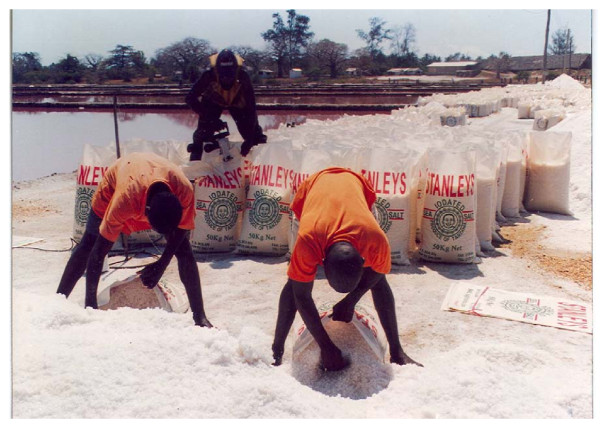
**After iodation the process of packaging salt from the heap results in further mixing of salt with iodate and the potential to improve homogeneity**.

Spraying the salt on a heap while shovelling (Figure 2) or on a mat/polyethylene mixing sheet (Figure 3) was regarded as critical for obtaining a homogeneous mixture of iodated salt; both types of sprayers were studied.

Two 50 kg bags of salt were spread on a polyethylene sheet at a thickness of 0.5 – 1.0 inches. After the salt was sprayed, it was mixed with a shovel (for heaps) or by curling the polyethylene sheet (or mat) and re-spreading the salt. This was repeated five times.

The iodine levels achieved using the diesel/petrol-powered cement mixer recommended as an iodate mixing machine [24] were compared with those achieved by the manual mixing method using the mat/polyethylene sheet. For all heaps studied, the workers used a cropper (a piece of flat plastic or wood) to package the salt into the fifty-kg bags (Figure 3).

The study team then trained the salt workers in the modified iodation and mixing procedures and in the handling and maintenance of the sprayers. To check whether the workers understood the new procedures, three trained salt workers were picked randomly from a group of ten to carry out the salt iodation exercise without supervision and their iodation performance was assessed. Randomly selected bags were sampled.

### Data analysis

Qualitative data from field notes were analysed, focusing on components that emerged as most relevant to the study [21]. Quantitative data were entered and analysed using Excel and Stata v 9.2. For each of the application and mixing methods used, the mean, standard deviation (SD), coefficient of variation and range of iodine concentrations were determined as well as the proportion of samples within the desirable range. Analysis of variance (ANOVA) was used to compare the performances of the different methods.

## Results

### Observations from salt works prior to the experiments

Salt workers reported that they currently had stocks of iodated salt. Iodation was reportedly done using a solution of potassium iodate (100 g KIO_3 _dissolved in 20 litres of water) although the container used in the large-scale factory site was found to have a 25 litre capacity. In observing the type of iodation equipment used and the process of salt iodation, a number of factors that contributed to low or inconsistent iodine levels were summarised by the study team prior to experimentation (Table 1).

**Table 1:** Summary of qualitative study findings

**Variables**	**Findings**
Location for salt iodation	- Done in open space, on different heap sizes (range 2–3 tons) at production sites to reduce transport costs and labour.
	- Iodation of salt was done at the sites; and iodated salt was loaded to trucks after iodation on sites.
	- All sites have storage facilities for salt in good production seasons or in case of unpredicted rains.

Salt iodation equipment	- Knapsack spray pumps were commonly used in all sites because they are simple and affordable. They can last for six months to one year depending on usage.
	- Conventional iodation machines e.g., cement mixers and machines with conveyors were found abandoned on sites due to high running costs and lack of spare parts.

Condition of knapsack spray pumps for salt iodation	- Some were rusty (metal components) and leaky (at the joints) resulting in wastage of iodated solution.
	- Some spray pumps were blocked by crusting in the sprayer nozzle, impairing the speed of spraying and mixing. Loss of iodated solution was also dependent on the wind direction.

Procedure for salt iodation and mixing	- At least two people were working at each heap; one spraying potassium iodate (KIO_3_) solution and one mixing salt with a shovel or spade.
	- Three iodation steps were observed i) making the iodate solution, which less than 100 g was dissolved in more than 20 litres, resulting in less iodine concentrations in iodated salt (10 g KIO_3_/litre of water is the correct concentration) ii) spraying and mixing not done adequately to acquire homogeneously mixed iodated salt, iii) packaging in 50 kg bags.
	- In some instances iodate solution was sprayed only on the top layer of salt in the upright filled bag without further subsequent mixing.

Workers knowledge on salt iodation	- Workers had minimum skills in salt iodation techniques and knowledge of iodation rationale.
	- Workers have shortage of weighing scales and were uncertain of the quantity of KIO_3 _required to deliver the recommended iodine levels 40 – 80 ppm.
	- Quantities of KIO_3 _and volumes of water varied from one producer to another. Sometimes brine (with high density) was used for dissolving KIO_3_, requiring more time to dissolve and possibly more pressure from the sprayer. Moisture content of iodated salt was measured by dry hand palm. The more the crystals of salt remains, the higher the moisture content in salt.

Supervision & monitoring	- Inadequate supervision (internal and external) to ensure quality control.
	- Records of quantities of salt produced monthly/annually were available but did not indicate whether iodated or not.
	- Field rapid test kits for iodated salt were adequately available in all sites but were not frequently used by workers to check the salt.
	- All necessary requirements for titration were observed in two sites, and reported that trained quality control personnel have left the salt business leaving no replacement and therefore the equipments were found not in use and/or damaged.
	- Salt workers were aware of salt testing by salt inspectors and the existence of salt regulations, therefore took effort to iodate top layers of the salt in bags to avoid troubles with enforcers.

Price of local iodation sprayers	Knapsack sprayer (20 litre capacity) costs Tanzanian Shillings 30,000 – 50,000 (equivalent US$ 25 – 40) and hand-bottle sprayers (one to two-litre capacity) cost Tanzania Shillings 6,000 – 10,000 (US$ 5.0 – 8.0).

Source of potassium iodate	Large salt producers import KIO_3 _from India costing about US$ 22 per kilogram including freight and custom duties. Others buy from the Tanzania Salt Producers Association (TASPA) or from their fellow producers at a price of Tanzania Shillings 25,000 – 30,000 (US$ 20 – 25) per Kg.

Shortage of iodation sprayers led workers to rush to share the sprayers in order to achieve their daily production quota, with minimum attention to the quality of iodation, contributing to an unsatisfactorily iodated salt product. The upright positioning of the salt bags with additional iodation of the top layer that we observed was later confirmed by the workers, who said: *"We normally do so in order to be sure in case some salt did not get enough iodine. We also believe *[probably more or less correctly]*that the sprayed solution will migrate down the bag reaching each salt particle, as long as bag does remain in that position"*. It was further learned from the workers' statements that the main reason for high iodine levels in the top layers was to satisfy the salt inspecting authorities (this salt is sometimes referred to in Swahili as "*Chumvi ya Bwana Afya*", meaning "salt for the health inspector"). Salt producers were aware that health inspectors regularly check salt consignments at different points during transportation and in shops using test kits, and that findings will always be confirmed by titration method before any legal action is taken against those found to contain less iodine than required.

The overall median iodine content of the salt from three heaps iodated 3 – 4 days prior to the arrival of the study team and sampled from packaged 50 kg bags was 10.6 (range 1.1 – 110.0 ppm for heaps 1 – 3, Table 2). The iodine levels were high in the top layers in the bags (mean 43.3 ppm ± 34.3SD) and very low in the middle (8.5 ppm ± 3.2SD) and bottom (6.8 ppm ± 5.0SD) layers. Only 9% of the 45 samples fell within the required range (40 – 80 ppm).

**Table 2:** Iodine levels in salt samples treated differently during iodation processes

Condition of salt treatment	Salt heap code no.	Iodine content in salt (ppm)							
		
		Top layer in bags		Middle layer in bags		Bottom layer in bags		Estimated total iodine	
		
		n	Median (range)	n	Median (range)	n	Median (range)	n	Median (range)
Unsupervised iodation without altering KIO3 concentration (<100 g) or equipment	1	5	19.0 (18.0 – 25.4)	5	10.6 (6.3 – 11.6)	5	8.5 (4.2 – 15.9)	15	11.6 (4.2 – 25.4)
	2	5	45.5 (2.1 – 60.3)	5	5.3 (4.2 – 6.3)	5	2.1 (1.1 – 5.3)	15	5.3 (1.1 – 60.3)
	3	5	97.3 (14.8 – 110)	5	10.6 (8.5 – 14.8)	5	5.3 (4.2 – 18.0)	15	12.7 (4.2 – 110.0)
	
	**Total**	**15**	**25.4 (2.1 – 110.0)**	**15**	**8.5 (4.2 – 14.8)**	**15**	**5.3 (1.1 – 18.0)**	**45**	**10.6 (1.1 – 110.0)**

Unsupervised iodation using improved method and 204 g KIO3	8	5	55.0 (43.4 – 61.4)	5	55.0 (41.3 – 65.6)	5	58.2 (32.4 – 63.5)	15	56.1 (32.8 – 65.6)
	9	5	54.0 (50.8 – 74.1)	5	63.5 (59.2 – 71.9)	5	63.5 (59.2 – 83.6)	15	63.5 (50.8 – 83.6)
	10	5	61.5 (56.0 – 71.6)	5	58.2 (49.2 – 68.9)	5	65.4 (59.2 – 72.4)	15	61.5 (49.2 – 72.3)
	
	**Total**	**15**	**59.2 (43.4 – 74.1)**	**15**	**60.3 (41.3 – 71.9)**	**15**	**60.3 (32.8 – 50.8)**	**45**	**59.9 (32.8 – 83.6)**

### Development of an improved procedure for iodation

Salt was sprayed and mixed with iodate solution of a known concentration (prepared before training) under supervision. Other factors, such as the condition of sprayers and the volume of water used, were unaltered. The resulting iodine concentrations were consistently lower than expected (mean 13.2 ppm (± 2.4 SD)) but relatively homogenous.

In subsequent experiments, three different quantities of iodine solution used on different heaps yielded the following concentrations: 102 g KIO_3 _(mean 30.0 ppm iodine ± 5.8SD in heap no. 5), 204 g KIO_3 _(mean 60.9 ppm iodine ± 7.4SD in heap no. 6) and 250 g KIO_3 _(mean 72.8 ppm iodine ± 10.9SD in heap no.7) for expected required values of 30.3, 60.5 and 74.4 ppm iodine, respectively. Therefore, 204 g KIO_3_/20 litres (10 g KIO_3_/l) was selected as the appropriate iodine solution.

We observed that KIO_3 _dissolved more rapidly in fresh than in salt water. The use of hand bottle sprayers was observed to cause pain in the user's hands over time, resulting in reduced usage. They are suitable when the quantity of salt to be iodated is less than one tonne. Knapsack sprayers act so rapidly that the salt can be sprayed as it is being shovelled; thus, one can achieve a reasonable degree of coverage of relatively large salt crystals with no additional effort. Knapsack sprayers with slit nozzles gave an even and more easily monitored coverage than those with round nozzles.

### Testing iodation performance with improved methods

Ten salt workers were trained in the use of knapsack sprayers. The overall median iodine content then achieved without supervision was 59.9 (range 32.8 – 83.6) ppm for heaps 8 – 10 (Table 2) with 96% of the samples (n = 45) falling within 40 – 80 ppm, compared to only 9% with the original methods.

**Figure 4:**
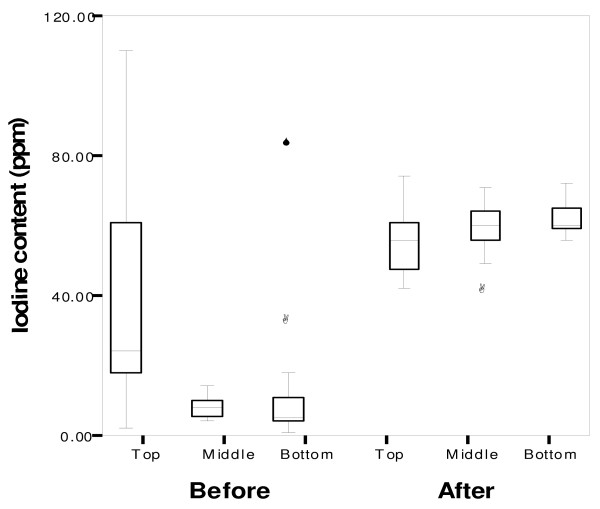
**Mean salt iodine concentration before and after modifications on top, middle and bottom layers of salt sacks**. NB: o = outlier, * = extreme outlier.

Figure 4 shows that (i) before training, the samples from the top layers of the bags had a higher iodine concentration and greater variability than those from the middle or bottom layers (p < 0.0001), and (ii) after training with improved methods, the mean iodine concentrations achieved in the middle and bottom layers were similar to those in the top, with no significant differences (p = 0.58). Thus, differences in iodine concentration in the salt samples from the three layers were very marked before the experiment and training but insignificant afterwards (p < 0.0001), reference iodine interval 40 – 80 ppm.

### Comparing mixing methods

**Figure 5:**
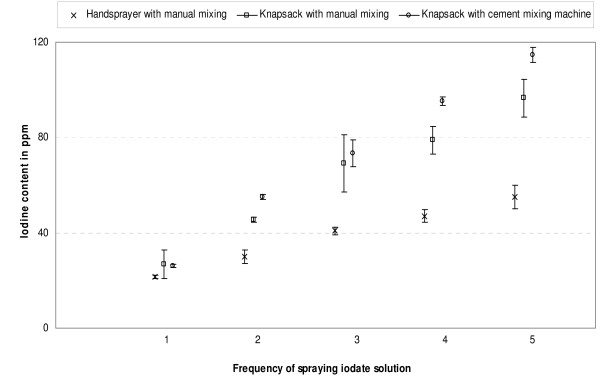
**Iodation performance of sprayers indicated by mean and standard deviation of concentrations achieved using manual and machine mixing of KIO_3 _solution and salt**. *NB: a shovel is used to mix the salt on heap while curling and re-spreading salt is used on mat/polyethylene sheet*.

Figure 5 shows the mean and SD of iodine concentrations achieved using hand and knapsack sprayers with manual and machine mixing. An increased number of sprayings of the same heap size with a hand sprayer failed to greatly increase the amount of iodine, probably because of fatigue. For the knapsack sprayer, iodine content increased approximately linearly with the number of sprayings for manual and cement mixing, the latter producing a small improvement (p = 0.05), and appeared greater at higher spraying frequencies.

Using the improved methods we found that a mean of 7.7 litres of iodate solution (from a stock of 204 gm KIO_3 _dissolved in 20 litres of water) can iodate one tonne of coarse salt (~2.6 tonnes/20 litres), taking an average of 10 minutes and yielding a final iodine concentration of about 60 ppm. Thus, results from three different heaps indicated that one kilogram of KIO_3 _can adequately iodate an average of 12.2 tonnes of salt (range 11.2 – 13.1 tonnes) to achieve the standard iodine range of 40 – 80 ppm.

## Discussion

We have demonstrated that locally adapted small-scale spraying technologies can yield adequate and homogenous iodate concentrations in salt in a low-resource setting if properly standardized and supervised. To our knowledge, this is the first study from Sub-Saharan Africa to assess the effectiveness of small scale salt producers' self-initiated salt iodation technology and to improve it to achieve both adequate and homogeneous iodine concentrations.

Standard operating procedures need to be developed and slight adaptations made to fit each producer's situation and needs. The salt workers in our study did not know the purpose of iodating salt for their customers and for themselves. They understood the existence of the salt law and probably feared its penalties [17], but not the consequences to consumers of inadequate or excessive iodine intake [8]. Owing to high staff turnover, training of salt workers needs to be ongoing, not just in the techniques but also in the rationale of salt-iodation. Routine supervision of the salt iodization procedure refers to the responsibility of foremen at production sites to oversee that the iodation procedure is adhered by salt workers. Failure to do so will result in high iodine variability and often failure to meet the required safe iodine levels.

To address this, greater levels of accuracy in salt testing need to be achieved using the titration method at larger facilities [18,23]. Therefore the national IDD programme needs to ensure that the revival of the mini-laboratories for quantitative analysis of iodine by titration method at production sites is accompanied by external supervision and repeat training of new staff. The Tanzania Bureau of Standards and enforcement bodies (Tanzania Food and Drugs Authority and Ministry of Energy and Minerals) [13] need also to work closely with salt producers (providing external quality control) to ensure that recommended iodine levels are met by the improved iodation method. The continuous implementation of quality control by factory management and oversight by the National IDD Control Program, the national food safety and standard regulating bodies, are important as reported elsewhere, where a well-monitored salt iodization program has resulted in a marked reduction in goitre prevalence and eventual normalization of urinary iodine concentrations [25].

The Tanzanian IDD Control Program previously considered iodation of salt by manual spraying and mixing methods to be inappropriate, but the strategy of providing machine-operated salt-iodation machines to salt producers in Tanzania has been unsuccessful in the long run [20]. Nevertheless, despite failure to use iodation machines and/or breakdowns, the producers continued to manufacture iodated salt using alternative self-initiated technologies and maintained a reasonable percentage coverage of iodated salt at household level [17,18]. They adopted low-cost low-technology spray-on approaches, as recently noted in Indonesia [26] and in Mozambique [18]. However, these methods were unable to yield the recommended iodation levels, entailing risks of either hypothyroidism or hyperthyroidism [12,13,20,27].

The low variability in the levels measured in different parts of the packaged bags from heaps iodated using the improved methods in our study indicated good short-term adherence, but an effective monitoring system may be required to maintain this [24,28]. Currently, the only widely applicable method for ensuring the presence of iodine in salt at production level in Tanzania is a qualitative method using RTK [22]. It should be feasible to provide at least large/medium-scale salt producers with mini-laboratories for quantitative analysis of iodine levels [7]. The programme did this in fact, but close follow-up is needed from national level to ensure their usage and technical support where weaknesses may exist, especially staff turnover, which commonly leaves facilities with no staff capable of using the equipment properly [20]. Table 3 proposes practices for salt production at various scales of operation.

**Table 3:** Proposed practices for salt-iodation at different scales of salt production

**Practice**	**Large scale salt producer**	**Medium scale salt producer**	**Small scale salt producer**
Quantity to iodate	Above 100 tons/day	1 – 100 tons/day	Below 1000 kgs/day

Type of iodating equipment/utensils	Conventional salt iodating machines	Knapsack sprayers with slit nozzles	Knapsacks if externally provided, otherwise 1 – 2 litre hand bottle sprayers

Iodation procedure	Machine calibrated spraying with automated mixing of salt	Hand pump spraying with manual shovel mixing or cement mixer	Hand pump spraying with manual mixing on mat

Amount of KIO_3 _used	76.5 g/ton	20 litres of solution (10 g/l) to iodate an average heap (2.5 tons)	Solution of 20 g in 2 litres to iodate an average of five 50 kg bags

Frequency of spraying to achieve recommended levels	Machine calibrated and controlled	2 – 4 times with manual mixing and 2 – 3 times with concrete mixer	4 – 5 times with hand bottle sprayers and manual mixing on mat

Homogeneity	Excellent	Good	Fairly good

Quality control	Laboratory control (both RTK and titration methods)	Laboratory control (both RTK and titration methods)	Semi quantitative method (RTK)

Frequency of internal quality control to determine magnitude of iodine variability*	Periodic validation of iodation processing and batch to batch iodine testing	Daily/periodic validation of iodation processing and batch to batch iodine testing	Daily validation of iodation processing and batch to batch iodine testing

NB * acceptable magnitude of variability is when the variation of the iodine content of all samples within and between batch(es) is less than 2% from the target value (e.g., 100 ppm) and the relative standard deviation (RSD) is equal to or less than 3%, then the potassium iodate/salt mixing is considered homogeneous [24].

Difficult field conditions constrain the workings of these salt factories and similarly constrained the conduct of this study. For example, relatively small samples had to be used in estimating the total amounts of iodine present in 50 kg bags of salt and heaps of salt that weighed 2–3 tonnes.

Lack of data on moisture content and impurities in the salt before and after iodation was also a limitation in this study. These factors can influence iodine losses during storage, salt as reported elsewhere [19]. As an informal test method for moisture content, salt workers were observed to take salt and hold in dry hands. If not many crystals remained on their palms, this was taken as an indication that the salt had low moisture content. Previous reported findings of moisture by this informal test have been similar to those obtained by analytical method and the salt we observed did meet Tanzania's recommended standards [20].

Current salt iodation strategies fail to reach 30% of the population [29], so alternative strategies are required to achieve WHO's over 90% coverage goal [8]. Policymakers need to work with the small-scale producers who generate employment for thousands of workers in isolated areas[30] while continuing to protect iodine nutrition in all population groups [18,31-33].

## Conclusion

In summary, with standardized procedures, close supervision of the improved iodation procedures, and testing of the end product by the site quality control foreman and national programme and standard bureau bodies, self-initiated spray methods of salt iodation can yield adequate and homogenous iodine levels in low-resource settings. Coupled with continuous training, monitoring and enforcement, this is a promising approach for including small-scale salt producers in the drive to achieve universal salt iodation and eliminate IDD.

## Competing interests

The authors declare that they have no competing interests.

## Authors' contributions

VDA, SP, MR, PBM, TG contributed to the study concept, design and drafted the manuscript. PBM, VDA, MM, and NVM contributed in acquisition of data. VDA, MR and SP contributed in statistical analysis. TT, TG, SP, VDA provided critical revision of the manuscript for important intellectual content. All authors participated in the interpretation of the results and have seen and approved the final version.

## Pre-publication history

The pre-publication history for this paper can be accessed here:



## References

[B1] De Benoist B, Andersen M, Egli I, Takkouche B, Allen H, WHO (2004). Iodine Status Worldwide. WHO Global Database on Iodine Deficiency.

[B2] Andersson M, Takkouche B, Egli I, Allen H, de Benoist B (2005). Current global iodine status and progress over the last decade towards the elimination of iodine deficiency. Bulletin of the World Health Organisation.

[B3] UNICEF (Ed) (2007). Progress For Children: A World Fit for Children Statistical Review.

[B4] Hetzel BS (2005). Towards the global elimination of brain damage due to iodine deficiency: The role of the International Council for Control of Iodine Deficiency Disorders. International Journal of Epidemiology.

[B5] UNICEF (2008). Protecting children's brain development through universal salt iodization: Successes and Challenges in Eastern and Southern Africa.

[B6] UNICEF-WHO (1994). World Summit for Children – Mid-Decade Goal: Iodine Deficiency Disorders (IDD). Joint Committee on Health Policy, Special Session, Agenda Item 2 2 7.

[B7] Mannar V, Dunn J, ICCIDD (1995). Salt iodization for elimination of iodine deficiency.

[B8] WHO/UNICEF/ICCIDD (2007). Assessment of iodine deficiency disorders and monitoring their elimination: A guide for programme managers.

[B9] Haar F van der, Kavishe P, Medhin M (1988). The public health importance of IDD in Tanzania. Cent Afr J Med.

[B10] Wachter W, Mvungi M, Konig A, Pickardt C, Scriba P (1986). Prevalence of goitre and hypothyroidism in Southern Tanzania: effect of iodised oil on thyroid hormone deficiency. J Epidemiol Community Health.

[B11] Kavishe F, Mushi S (1993). Nutrition Relevant Actions in Tanzania A case study for the XV Congress of the International Union of Nutrition Sciences.

[B12] Assey V, Mgoba C, Mlingi N, Sanga A, Ndossi G, Greiner T, Peterson S (2007). Remaining challenges in Tanzania's efforts to eliminate iodine deficiency. Public Health Nutrition.

[B13] The United Republic of Tanzania (1994). Salt Acts: The Mining Act 1979: The mining (salt production and iodation) regulations 1994 and The Food (Control of Quality) Act 1978. Regulations made under section 16 (1) and (2). The Food (Iodated salt) regulation. Salt Acts. Dar es Salaam, Tanzania 1994. Government gazette.

[B14] WHO/ICCIDD/UNICEF (1997). Review of findings from seven-country study in Africa on levels of salt iodization in relation to iodine deficiency disorders including iodine induced hyperthyroidism.

[B15] SCN (2004). 5th Report on the World Nutrition Situation: Nutrition for Improved Development Outcomes.

[B16] National Bureau Statistics (NBS) [Tanzania] (2005). Tanzania Demographic Health Surveys 2004–05.

[B17] The United Republic of Tanzania (2006). The Tanzania Food, Drugs and Cosmetics (Iodated Salt) Regulation 2006, Made under section 122(1), c.

[B18] UNICEF (2007). Protecting Children's Brain Development: Strategic review on sustained universal salt iodization in Eastern and Southern Africa. Report of a workshop 25–26 April 2005.

[B19] Diosady L, Alberti M, Mannar V, Stone T (1997). Stability of iodine in iodized salt used for correction of iodine-deficiency disorders. Food & Nutr Bull.

[B20] Assey VD, Peterson S, Greiner T (2008). Sustainable universal salt iodization in low-income countries – time to re-think strategies?. European Journal of Clinical Nutrition.

[B21] Dahlgren, Emmelin M, Winkvist A, (Eds) (2004). Qualitative Methodology for International Public Health.

[B22] Diosady L, Mannar V, Geertman R (2000). Development of rapid test kits for monitoring salt iodization. 8th World symposium, conference.

[B23] Sullivan K, Houston R, Cervinskas J, Gorstein J, UNICEF/PAMM/MI/ICCIDD/WHO (1995). Titration methods for salt iodine analysis. Monitoring universal salt iodization.

[B24] Sullivan K, Houston R, Cervinskas J, Gorstein J, UNICEF/PAMM/MI/ICCIDD/WHO (1995). Monitoring universal salt iodization programs.

[B25] Azizi F, L M, Sheikholeslam R, Ordookhani A, Naghavi M, Hedayati M, Padyab M, Mirmiran P (2008). Sustainability of a well-monitored salt iodization program in Iran: marked reduction in goiter prevalence and eventual normalization of urinary iodine concentrations without alteration in iodine content of salt. J Endocrinol Invest.

[B26] Tanduk T, Wahjono S, Hernanto B, Marihati, Fahmida U, Agustina R (2006). Study report:Salt Iodization Using Hand Spraying in Indonesia: A Feasibility Study Report submitted to UNICEF by the Ministry of Industry and SEAMEO-TROP-MED RCCN University of Indonesia. ICCIDD Newsletter.

[B27] WHO/ICCIDD/UNICEF (Ed) (1996). Recommended iodine levels in salt and guidelines for monitoring adequacy and effectiveness Volume WHO/NUT/9613.

[B28] Azizi F (2003). Salt Iodization, Monitoring, and Evaluation (SIME): an Effective Replacement for Universal Salt Iodization (USI). International Journal of Endocrinology Metabolism.

[B29] UNICEF (Ed) (2007). The State of the World's Children 2007.

[B30] ICCIDD, MI (2007). Supporting small scale salt producers is essential for achieving USI. IDD Newsletter.

[B31] Sundqvist J, Wijetunga M, Assey V, Gebre-Medhin M, Peterson S (1998). Salt iodation and risk of neonatal brain damage. Lancet.

[B32] Lancet (2008). Iodine deficiency- way to go yet. The Lancet.

[B33] Zimmermann M, Jooste P, Pandav C (2008). Iodine-deficiency disorders. The Lancet.

